# The preparation and characterization of folate-conjugated human serum albumin magnetic cisplatin nanoparticles^☆^

**DOI:** 10.1016/S1674-8301(10)60005-X

**Published:** 2010-01

**Authors:** Daozhen Chen, Qiusha Tang, Wenqun Xue, Jingying Xiang, Li Zhang, Xinru Wang

**Affiliations:** a Key Laboratory of Reproductive Medicine, Institute of Toxicology, Nanjing Medical University, Nanjing, 210042, China; b Clinical Laboratory ,Wuxi Hospital for Maternal and Child Health Care Affiliated Nanjing Medical University, Wuxi, Jiangsu,214002, China; c Medical School Southeast University, Nanjing, Jiangsu, 210009, China.

**Keywords:** cisplatin, folate, albumin magnetic nanoparticles, conjugate

## Abstract

**Objective:**

Nanoparticles are becoming an important method of targeted drug delivery. To evaluate the importance of folate-conjugated human serum albumin (HSA) magnetic nanoparticles (Folate-CDDP/HSA MNP), we prepared drug-loaded Folate-CDDP/HSA MNPs and characterized their features.

**Methods:**

First, folate was conjugated with HSA under the effect of a condensing agent, and the conjugating rate was evaluated by a colorimetric method using 2, 4, 6 - trinitrobenzene sulfonic acid. Second, under N_2_ gas, Fe_3_O_4_ magnetic nanomaterials were prepared and characterized by using transmission electron microscopy (TEM), SEM-EDS and X-ray diffraction (XRD). Finally, Folate-CDDP/HSA MNP was prepared by using a solvent evaporation technique. TEM was used to observe particle morphology. The particle size and distribution of the prepared complexes were determined by a Laser particle size analyzer. Drug loading volume and drug release were investigated by a high performance liquid chromatography method (HPLC) *in vitro*.

**Results:**

We successfully prepared folate-conjugated HSA and its conjugating rate was 27.26 µg/mg. Under TEM, Fe_3_O_4_ magnetic nanoparticles were highly electron density and had an even size distribution in the range of 10-20 nm. It was confirmed by SEM-EDS and XRD that Fe_3_O_4_ magnetic nanoparticles had been successfully prepared. Under TEM, drug-loaded magnetic nanoparticles were observed, which had a round shape, similar uniform size and smooth surface. Their average size was 79 nm which was determined by laser scattering, and they exhibited magnetic responsiveness. Encapsulation efficiency was 89.75% and effective drug loading was calculated to be 15.25%. The release results *in vitro* showed that the half release time (t½) of cisplatin in cisplatin Solution and Folate-CDDP/HSA MNP was 65 min and 24 h respectively, which indicated that microspheres had an obvious effect of sustained-release.

**Conclusion:**

Folate-CDDP/HSA MNPs were prepared successfully. The preparation process and related characteristics data provided a foundation for further study, including the mechanism of the nanoparticles distribution *in vivo* and their intake by tumor cells.

## INTRODUCTION

Magnetic drug loaded nanoparticles are an ideal type of formulation for targeting drugs for chemotherapy. The drug distribution of such a formulation can be changed by applying a magnetic field, concentrating the drug at the tumor sites. Such formulations can also delay drug release and reduce any toxic effect of the drug. The formulation can be designed to use receptors as targets, producing a drug delivery system that is specifically targeted. Among the various nanoparticle colloidal systems, those based on proteins may be particularly promising because of their biodegradability, lack of toxicity and antigenicity, stability, and shelf life, providing controllable drug-release properties and high loading capacity for hydrophilic molecules[1].

To solve the problem of site-specific targeting for the colloidal systems, some authors have attempted to increase the tissue specificity of colloidal drug carriers by coupling targeting agents. Among the possible targeting agents, folic acid could be exploited to realize delivering drugs into cancer cells. Folic acid is a low molecular weight (441 Da) vitamin whose receptor is frequently overexpressed in human cancer cells. This receptor has been identified as a tumor marker, especially in ovarian carcinomas, and it is highly restricted in most normal tissues[2].

In the present study, we first conjugated folate with human serum albumin (HSA) to prepare folate-conjugated HSA, then made use of its capsule to encapsulate magnetic nanoparticles and cisplatin to prepare folate-conjugated magnetic cisplatin nanoparticles. The preparation process and related characteristics were investigated, and these will be used to lay the foundation for further study, including determining the mechanism of the nanoparticles intake by tumor cells and their distribution *in vivo*.

## MATERIALS AND METHODS

### Instruments

Startorious 17-1 type electronic balance (Germany), UV-2201 spectrophotometer (Shimadzu, Japan), Malvern-2000 Laser Scattering particle size analyzer (UK), HITACHI H-600 transmission electronic microscope (TEM) (Japan), CQ50 ultrasonic cleaner (Shanghai Ultrasonic Instrument Factory, China) and X-ray diffraction (XRD) (Shimadzu, Japan).

### Agents and reagents

HSA (Shanghai Shenggong Biotech Co., Ltd., China), Sephadex G-250 (Pharmacia), EDC (Shanghai Sanjie Bio-tech Co., Ltd., China), folic acid (Sigma-Aldrich, USA), trypsase (Acros Organics, USA), cisplatin for injection (Shandong Qilu Pharmaceutical Co., Ltd., China), standardized cisplatin (China Pharmaceutical Bio-products Evaluation Institute, China), and the other reagents were analytically pure.

### Preparation and characterization of folate-conjugated human serum albumin (Folate-HSA)

Folic acid (30 mg) was dissolved in 1,000 µl phosphate buffer solution (pH 9.0). After the folate was completely dissolved, EDC was added to the folate solution at the molar ratio of 6:1, mixed fully and activated for 15 min. Then 5 ml PBS solution (pH 9.0) of 50 mg/ml HSA was added into the above solution, and allowed to react with each other for 2 h while stirring. The above reaction solution was separated through a Sephadex G-250 dextran gel column, and the light yellow opalescent mobile phase was collected. The light yellow opalescence was due to the Folate-HSA. The degree of conjugation of the Folate-HSA was calculated as described elsewhere[3].

### Preparation and characterization of magnetic material

#### Preparation of magnetic material

A previously described method was used[4]. Under N_2_ gas, FeCl_3_•6H_2_O and FeCl_2_•4H_2_O were dissolved in deionized water at a ratio of n(Fe^2+^) : n(Fe^3+^)=2:1, by magnetic mixing. Ammonia water (0.4M) was then slowly dripped into the molysite solution, which was hydrolyzed, generating a large number of black crystal particles. The ammonia water was then dripped into the above solution again, and the pH adjusted to make the solution alkaline. The material was aged at 80°C in a water bath, magnetically separated, and then washed several times by centrifugation. The wet precipitate was put into an acid solution, and finally a brown colloidal sol was obtained by peptization at 60°C.

#### Characterization of magnetic material

Morphology of nanoparticles was observed by TEM, and the nanoparticle physical properties studied with XRD (analytical conditions: Cu target; testing voltage: 40kV; current: 30mA; λ = 1.542), The nanoparticle composition was analyzed by SEM-EDS (testing conditions: accelerating voltage: 15KeV; take-off angle: 25.693°; live time: 210 seconds; dead time: 13.658 seconds).

### Preparation and characterization of folate-conjugated human serum albumin magnetic cisplatin nanoparticles (Folate-CDDP/HSA MNPs)

#### Preparation of Folate-CDDP/HSA MNPs

As previously described[5], 250 mg Folate-HSA, 100 mg magnetic powder and 25 mg cisplatin were mixed thoroughly, and the volume adjusted to 25 ml and the pH to 9.0; next, 150 ml anhydrous alcohol was slowly added at room temperature (1 ml/min), and the mixture was stirred for half an hour, at which time the solution appears hazy. 50 µl glutaraldehyde was added slowly with continuous stirring and cured for 12 h. The reaction solution was centrifuged at 7 100 g, and the precipitate washed three times with water, and dried under vacuum. Finally, reserve samples were maintained as described below.

#### Magnetic test of prepared Folate-CDDP/HSA MNPs

As described elswhere[6], nanoparticles were added into normal saline (10 mg/ml), with ultrasonic mixing. Then one drop of nanoparticles was placed onto a slide and observed with an inverted microscope. Nanoparticles were uniformly distributed in the solution. Then a permanent magnet with a surface magnetic field strength of 5 000 gauss was placed on one side of the drop on the slide, and the motion of the nanoparticles was observed microscopically.

#### Morphological observation of prepared Folate-CDDP/HSA MNPs

A thoroughly mixed suspension of prepared Folate-CDDP/HSA MNPs was taken and dripped on to a copper mesh EM grid, negatively stain with 2% phosphotungstic acid at room temperature, and the nanoparticle morphology observed and imaged with a HITACHI-600 transmission electron microscope.

#### Particle size analysis of Folate-CDDP/HSA MNPs

A thoroughly mixed suspension of prepared Folate-CDDP/HSA MNPs was added to 100 ml distilled water, and the particle size distribution of the nanoparticles was determined with a laser particle size analyzer.

#### Testing of drug loading volume and in vitro drug release property

##### Standard curve

Cisplatin standard solutions were obtained by diluting a 0.04 g/L aqueous CDDP solution to make standards of 10, 20, 30, 40, 50 and 60 µg/ml. 20 µl of the standard solutions was injected into the high performance liquid chromatograph (HPLC) and linear regression was used to fit a standard curve of area (A) vs concentration (C). Chromatographic conditions: mobile phase, methanol: 0.9% sodium chloride solution (80:20, V/V); flow rate: 1.0 ml/min, sampling volume: 20 µl, test wave length 310 nm.

##### Test of drug loading volume

The Folate-CDDP/HSA MNPs were ultrasonically homogenized in saline and then dispersed in 0.5% pepsin solution (or washed three times by centrifugation), digested in a water bath at 37±1°C for 2 h, and then centrifuged at 25 000 g for 20 min. The supernatant fluid was taken and diluted, and 20 µl supernatant fluid used for HPLC determination.The sample cisplatin content was calculated, and the drug loading volume and encapsulating rate were determined using the following formulae: drug loading volume = cisplatin content in albumin nanoparticles/weight of albumin nanoparticles×100%; encapsulation rate = cisplatin content in albumin nanoparticles/original dose×100%

##### Investigation of in vitro drug release

Magnetic cisplatin albumin microspheres (60 mg) were added to normal saline, then ultrasonically homogenized, centrifuged, dispersed in a final volume of 4 ml normal saline, and then transferred to a dialysis bag. The bag was suspended in a conical bottle with 25 ml normal saline with constant-rate stirring (150 r/min) at (37±1)°C. Samples (3 ml) were periodically taken and a corresponding volume replaced. HPLC was used to determine the CDDP concentration and calculate the drug release volume and accumulated drug release fraction using a regression equation, and an *in vitro* drug release curve was obtained. The release of CDDP from raw cisplatin solution was similarly investigated.

### Statistical analysis

The statistical analysis was performed by using SPSS software (Version 16.0, SPSS Inc., USA). The model fitting of the nanoparticle drug release behavior *in vitro* pharmacokinetics was calculated by 3p87 software (Chinese Pharmacologic Society).

## RESULTS

### Degree of conjugation of Folate-HSA

**Fig. 1:**
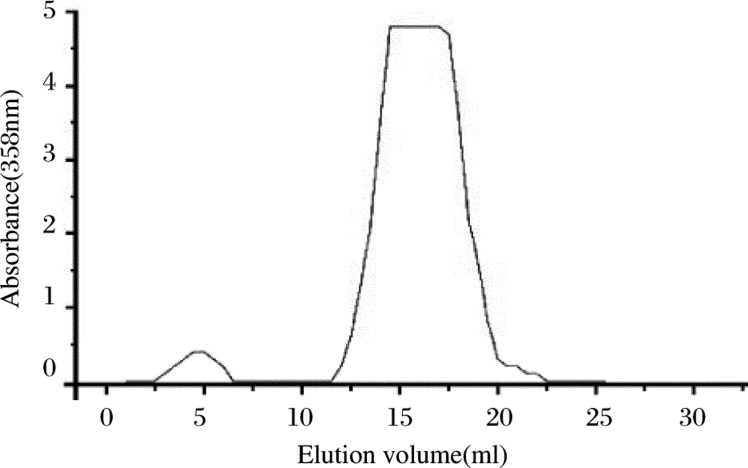
Folate-HSA ultraviolet elution curve

The Folate-HSA conjugate was prepared successfully ***(Fig. 1)***. The average folate conjugated with HSA was 45.8 µg/mg HSA, and the percentage of the folate that conjugated on the surface of HSA nanoparticles was 27.26%.

The ultraviolet elution curve of the Folate-HSA passed through a Sephadex-G250 dextran gel column ***(Fig. 1)*** showed two distinct peaks, the first peak corresponding to conjugated Folate-HSA, and the second peak corresponding to non-reacted folate. As shown in ***Fig. 1***, Folate-HSA was completely separated from non-reacted folate and other impurities.

### Characterization of magnetic material

#### Morphological observation of Fe_3_O_4_ magnetic nanoparticles

**Fig. 2:**
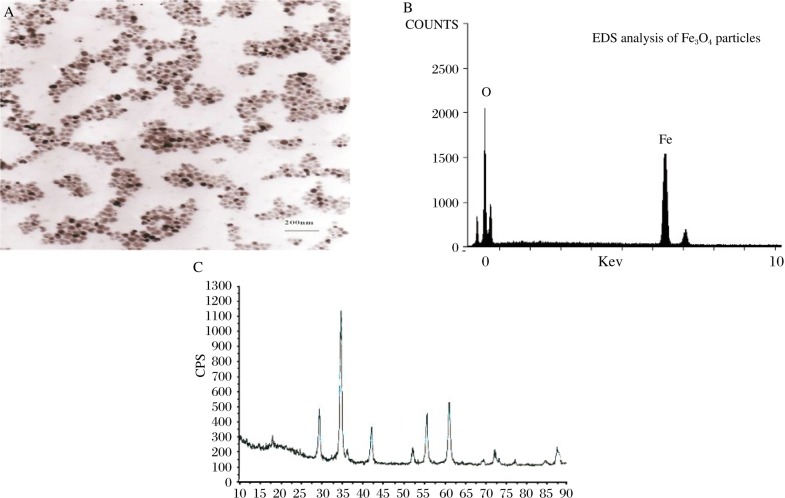
Fe_3_O_4_ magnetie nanoparticles detected by TEM(A), SEM-EDS(B) and ×RD(C)

Fe_3_O_4_ magnetic nanoparticles exhibited high electron density and a round shape under TEM ***(Fig. 2A)***. Some nanoparticles aggregated, but their size was uniform with diameters in the range of 10-20 nm.

#### Surface energy spectrum analysis of Fe_3_O_4_ magnetic nanoparticles

SEM-EDS analysis is shown in ***Fig. 2B***: the percentages of iron and oxygen in Fe_3_O_4_ magnetic nanoparticles were 73.77% and 26.23% respectively, which were consistent with those in Fe_3_O_4_. The finding confirmed the successful preparation of Fe_3_O_4_.

#### Physical phase analysis

X-ray diffraction ***(Fig. 2C)*** of prepared ferrite magnetic nanoparticles showed sharp diffraction peak, demonstrating good crystallization. Face distance (d value) corresponding to each diffraction peak coincides with that of 19-629 of powder diffraction file (PDF) (the code represents spinel magnetic Fe_3_O_4_) compiled by JCPDS, demonstrating that our experiment had successfully prepared spinel Fe_3_O_4_ magnetic nanoparticles.

### Characterization of Folate-CDDP/HSA MNP

**Fig. 3:**
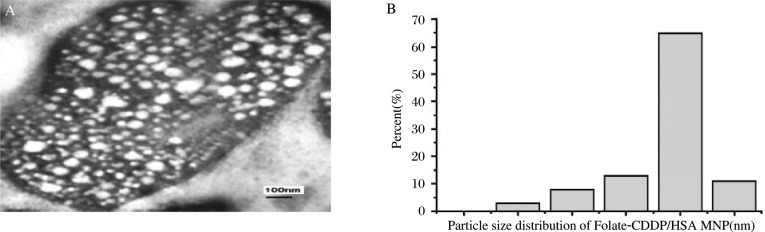
TEM (A) and particle size distribution(B) of Folate-CDDP/HSA MNPs

Under TEM, nanoparticles were found to have a round shape, uniform size and smooth surface ***(Fig.3A)*** The TEM results analysed by the laser particle size analyzer are shown in ***Fig. 3B***: the maximum particle size was 140 nm and minimum particle size was 30 nm, while 80% of microspheres were between 60-90 nm, and showed good dispersity. Their average size was 79±8.6 nm (kCounts:423.6). In a magnetic test, when a magnet was placed beside the slide, the nanoparticles moved towards the magnet and aggregated at that side within a few minutes.

### Test results of drug loading volume and *in vitro* drug release

The absorbance (A) vs cisplatin concentration (C) linear regression equation was, A=2.21387C-1.4037, *r* = 0.9999. The absorbance curve was linear over the concentration range 1-60 µg/ml. The enzyme digestion product value of Folate-CDDP/HSA MNPs was put into the regression equation to obtain the drug loading volume and envelopment rate. The envelopment rate was 89.75% when determined directly from Folate-CDDP/HSA MNPs, and the effective drug loading was 15.25% when the drug was washed away from the surface of the Folate-CDDP/HSA MNPs by stirring in normal saline.

**Fig. 4:**
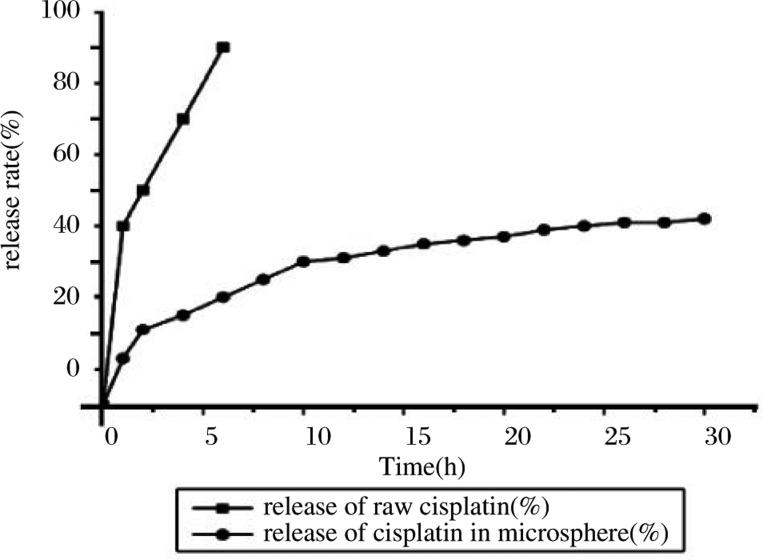
*In vitro* cisplatin release curve of Folate-CDDP/HSA MNPs

***Fig. 4*** shows the *in vitro* release of cisplatin. The release rate from Folate-CDDP/HSA MNPs was significantly slower than that in pure cisplatin solution, and it became slower with increasing time. The half release times (t½) of cisplatin in cisplatin solution and Folate-CDDP/HSA MNPs were 65 min and 24 h respectively. The model fitting of the drug release behavior *in vitro* was analyzed by zero-order kinetics equation, a kinetic equation, Higuchi equation, and Weibull equation. The results are shown in ***Table 1***. The model of adriamycin coupled albumin nanoparticles release *in vitro* fit the Weibull equation, expressed as ln[-ln(1-Q)]=0.3485lnt-3.1054, and the correlation coefficient was 0.9810. The above data indicated that microspheres have a significant effect on sustained release.

**Table 1:** The regression equation of Adriamycin release *in vitro*

Model	Regression equation	r	R^2^
Higuchi	Q=0.0141t½+0.0611	0.9548	0.9116
Zero order	Q=0.0009t+0.0915	0.9125	0.8327
Frist order	Ln(1-Q)=-0.001t-0.0974	0.9142	0.8358
Weibull	Ln[-ln(1-Q)]=0.3485lnt-3.1054	0.9810	0.9624

## DISCUSSION

The search for an effective drug target delivery system is a hot topic in tumor bio-therapy. Therapy that combines a receptor and its ligand has speciticity, selectivity, saturation, strong affinity and a high probability of being bio-effect. A receptor-mediated drug delivery system makes use of specific receptors on some tissues, or over-expressed receptors of tumor cells to transfer drug to the target tissues and into the cells by pinocytosis. High specificity and high affinity can greatly improve drug delivery efficiency, increase focused drug concentration and therapeutic effect, reduce toxic effects and achieve the desired targeted treatment. For these reasons, targeted drug delivery is currently one of the most active research fields[3],[5]. Many studies have found that the activity and quantity of folate receptors on the surface of tumor cells (such as cancer of the ovary, colon and rectum, breast, lung and renal cells) are significantly higher than those on normal cells. With a greater understanding of cell surface folate receptors, the study of folate mediated drug targeting of tumor cells has attracted more attention[1].

In the present study, we first used EDC to activate folate, and then conjugated the activated folate with HSA. A Sephadex G-250 column was used after conjugation, because the molecular weight of folic acid is far less than that of HSA[3],[5]. ***Fig. 1*** shows the UV elution curve of Folate-HSA through a Sephadex G-250 dextran gel column. The first peak corresponds to decontaminated Folate-HSA, and the second peak corresponds to non-reacted folate. Thus Folate-HSA can be completely separated from non-reacted folate and other impurities. The present study selected albumin nanoparticles as the vector to encapsulate or adsorb drug, because albumin nanoparticle vectors have high target orientation, can control drug release, increase solubility and absorption rate of insoluble drugs, improve therapeutic effect and reduce toxic effects[7]. HSA is a safe and non-toxic carrier that is unlikely to cause any adverse immune reaction. In addition, it is bio-degradable and can be degraded *in vivo* by pancreatic proteolytic enzymes. Combining a drug with albumin can prevent its release at the injection site and enables the drug to be released slowly[8].

We used folate-conjugated HSA as the capsule to envelop the drug and magnetic nanoparticles to manipulate targeting. There are three main methods to prepare albumin nanoparticles: emulsion and curing, desolvation[9],[10] and polymer dispersion[11]. The present experiment adopted desolvation. The desolvation-chemical crosslinking method can keep drug activity while enveloping the drug in the nanoparticle. We adopted this method for further study[3],[5]. We then prepared Fe_3_O_4_ with superparamagnetism to form a magnetic drug delivery system which was loaded into albumin microspheres together with cisplatin. In the Investigation of *in vitro* drug release, the accumulative *in vitro* release behavior of cisplatin from Folate-CDDP/HAS MNPs was slow and there were no bursts of release, while that of adriamycin from Folate/HSA MNPs was in accordance with the Weibull equation (ln[-ln(1-Q)]=0.3485lnt-3.1054) with a high correlation coefficient (*r* = 0.981).

In future studies, rats can be used for the study of *in vivo* pharmacokinetics and body distribution. After the administration of CDDP solution and nanoparticles, a RP-HPLC method with ultraviolet detection system could be developed for the determination of CDDP in rat plasma and tissues at different times, and the pharmacokinetic parameters could be calculated by 3p87 software. The study of pharmacokinetics and body distribution is the obvious, important next step to evaluate targeting of Folate-CDDP/HSA MNPs.

Guided by an applied static magnetic field, magnetic microspheres administered locally or intravenously can be selectively concentrated at target tissue, organs, or tumor cell masses. The target drug delivery system has attracted wide attention. In an alternating magnetic field, magnetic materials can absorb the energy of electromagnetic waves and generate heat, and control the localized temperature within the range of 42-46°C to kill tumor cells.[12] The duration of the increased temperature can be adjusted to destroy cancerous tissues; while preserving normal tissues beyond the target. This treatment method is called magnetofluid thermotherapy.

In short, compared with related domestic and foreign studies, in our present study we prepared a new type of carrier for targeted drug delivery, which used magnetic albumin nanospheres as a carrier and folate receptor as target, combining chemotherapy with thermotherapy. Specifically we used high affinity bio-targeting of the target cell folate receptor with the nanoparticle ligand to guarantee safety: the therapeutic effect of this delivery system combines the oriented killing effect of chemotherapy with the physiotherapy effect of a magnetic induction temperature rise. This system can satisfy the criteria of safety, effectiveness and specificity. These three aspects must be met before a new type of biotherapy can be applied clinically.
